# Low prevalence of hepatitis B virus infection in HIV-uninfected pregnant women in Cape Town, South Africa: implications for oral pre-exposure prophylaxis roll out

**DOI:** 10.1186/s12879-022-07697-5

**Published:** 2022-09-01

**Authors:** Dvora Joseph Davey, Nei-yuan Hsiao, C. Wendy Spearman, Mark Sonderup, Nai-Chung Hu, Nyiko Mashele, Rufaro Mvududu, Landon Myer

**Affiliations:** 1 Department of Epidemiology, Fielding School of Public Health, University of California Los Angeles, 615 E Charles Young Drive S, Los Angeles, CA 90095 USA; 2 Division of Epidemiology and Biostatistics, School of Public Health, University of Cape Town, Cape Town, South Africa; 3 Division of Infectious Diseases, David Geffen School of Medicine, University of California Los Angeles, Los Angeles, CA USA; 4 Division of Medical Virology, Department of Pathology, University of Cape Town and National Health Laboratory Service, Cape Town, South Africa; 5 Division of Hepatology, Department of Medicine, Faculty of Health Sciences, University of Cape Town, Cape Town, South Africa

**Keywords:** Pre-exposure prophylaxis, Pregnant, Hepatitis B, HIV

## Abstract

**Background:**

Oral daily preexposure prophylaxis (PrEP) using emtricitabine (FTC)/tenofovir disoproxil fumarate (TDF) is recommended as standard of care for prevention in individuals at high risk for HIV infection, including pregnant and postpartum cisgender women. FTC/TDF is also active against hepatitis B virus (HBV); however, concern has been raised that providing PrEP to individuals infected with HBV could lead to hepatitis flares and liver injury, especially in the setting of suboptimal PrEP use.

**Methods:**

We conducted a cross-sectional analysis of baseline data from the PrEP in pregnant and postpartum women (PrEP-PP) cohort study from February 2020–March 2022 in one antenatal care clinic in Cape Town, South Africa (SA) to evaluate: (1) the field performance of a point of care test (POCT) (Determine II, Abbott Inc., Japan) for diagnosis of hepatitis B surface antigen (HBsAg) in a maternity setting, (2) the prevalence of HBV in a cohort of pregnant women not living with HIV.

**Results:**

We enrolled 1194 HIV sero-negative pregnant women at their first antenatal visit. Median age was 26 years (IQR = 22–31 years); 52% were born before 1995 (before universal HBV vaccination had started in South Africa). Median gestational age was 22 weeks (IQR = 16–30 weeks). There were 8 POCT and laboratory confirmed HBV cases among 1194 women. The overall prevalence of 0.67% (95% CI = 0.34–1.32%). In women born before 1995, 8 of 622 women were diagnosed with HBsAg; the prevalence was 1.29% (95% CI = 0.65–2.52%), and in women born in 1995 or after (n = 572); the prevalence was 0% (95% CI = 0.0–0.67%). We confirmed the test results in 99.8% of the rapid HBsAg (Determine II). Sensitivity was 100% (95% CI = 68–100%). Specificity was 100% (95% CI = 99.67–100%).

**Conclusion:**

The prevalence of HBV was very low in pregnant women not living with HIV and was only in women born before the HBV vaccine was included in the Expanded Program of Immunization. The Determine II POCT HBsAg showed excellent performance against the laboratory assay. HBV screening should not be a barrier to starting PrEP in the context of high HIV risk communities.

## Background

Hepatitis B virus (HBV) infection is a major public health problem in sub-Saharan Africa. The virus is highly endemic (≥ 8% HB surface antigen [HBsAg] prevalence) with an estimated 82 million people chronically infected in sub-Saharan Africa, representing one-quarter of the global pool of those with chronic HBV infection [1, 2]. In 1995, hepatitis B vaccination was incorporated into the Expanded Programme on Immunization for infants (at 6, 10, 14 weeks and 18 months) in South Africa [3]. Prior to vaccine introduction, South Africa was a country of overall high HBV endemicity with highest HBsAg seroprevalence documented in Black South Africans ranging from 5–16% in rural males, 8–9% in urban males, 4–12% in rural females, and 2.7–4% in urban females [2–7]. Chronic hepatitis B has decreased in prevalence from highly endemic levels prior to vaccine introduction to approximately 0.3% HBsAg seroprevalence rate in < 15 year oldsconfirming an impact of the vaccination program to date [3]. Despite this, South Africa has not yet met the world health organization (WHO) 2020 targets of a < 1% of HBsAg seroprevalence among children < 5 years old [8]. Data from a 2008 study, demonstrated that the prevalence of hepatitis B in people living with HIV in urban South Africa was 5 times greater than people who were not living with HIV [4]. A recent study in people receiving HIV care in South Africa found high incidence and persistence of HBV. The prevalence of hepatitis B surface antigen was 8.5% [95% confidence interval (CI): 7.7–9.3] at baseline and 9.4% (95% CI: 8.6–10.3%) at end of follow-up among people living with HIV [9].

Oral daily preexposure prophylaxis (PrEP) using emtricitabine (FTC)/tenofovir disoproxil fumarate (TDF) is recommended as standard of care for prevention in individuals at high risk for HIV infection, including pregnant and postpartum cisgender women [10–12]. Concern has been raised that providing PrEP to people living with HBV could lead to flare ups of HBV as both FTC and TDF have HBV antiviral activity. This is especially the case in people who start and stop PrEP, or have inconsistent PrEP use. As a result, clinical guidelines for PrEP recommend pre-enrollment screening included rapid testing for HBsAg.

Flares of HBV and clinical hepatitis have been reported in patients on treatment for chronic hepatitis B infection with tenofovir or those on TDF/FTC, following treatment withdrawal, including the post-partum period [13, 14]. In a study of maternal PrEP use in an antenatal clinic in Cape Town, South Africa, we used a rapid point of care (POCT) hepatitis B surface antigen test (HBsAg), the Determine HBsAg2 (Abbott Diagnostics Medical Co., Ltd., Japan). The objectives of the study were to: (1) evaluate the field performance of POCT for diagnosis of HBV at maternity setting, and (2) to evaluate the prevalence of HBV in a cohort of pregnant women not living with HIV.

## Methods

We conducted a cross-sectional analysis of baseline data from the PrEP in pregnant and postpartum women (PrEP-PP) cohort study from February 2020 to March 2022 in one antenatal care clinic in the Gugulethu Midwife Obstetrics Unit (MOU) in Cape Town, South Africa. Gugulethu’s population of 300,000 is predominantly of low socioeconomic status (48% unemployment; 64% of the adult population lives on ~ $35 per month). The population uses local public-sector health services that are provided free at the point of use. In 2018, the HIV prevalence among women attending the local antenatal care services provided by the MOU was 27% with > 80% of women living with HIV breastfeeding. In 2020, the Gugulethu MOU saw over 2000 new antenatal consultations per month, and 73% (n = 1460) were not living with HIV [15]. Trained study counselors conducted the HBsAg POCT according to manufacturer’s instructions. Study staff provided the client with the results on the same day within 15 min. The test result was recorded after 15 min as specified by the manufacturer and 30 min after to evaluate concordance to evaluate any changes in results in our tests validation. Additional blood sample were taken for all women for HBV surface antigen confirmatory testing using the Elecsys HBSAg II assay which is an electrochemiluminescence immunoassay using the sandwich principle (Roche diagnostics, Switzerland) at the National Health Laboratory Service reference laboratory in Groot Schuur Hospital, Cape Town, South Africa.

Study eligibility criteria included: (1) ≥ 16 years, (2) confirmed HIV-negative serostatus by a 4th generation antigen/antibody combination HIV test (Abbott Diagnostics Medical Co., Ltd., Japan), (3) confirmed pregnant (via ultrasound or urinary beta hCG), (4) intention to stay in Cape Town through the postpartum period, and (5) absence of contraindications to PrEP.

Women who tested positive with the HBsAg POCT were ineligible to participate in the PrEP clinical trial and were referred to the clinic for Hepatitis B birth dose vaccine and clinical management in line with national guidelines. In addition to the POCT, the study nurse collected intravenous blood that was sent to the national reference laboratory for confirmatory testing using the reference surface antigen test (RSAT) on serum.

## Results

We enrolled 1194 HIV sero-negative pregnant women at their first antenatal visit. Median age was 26 years (IQR = 22–31 years; range was 16–45 years old); 52% were born before 1995 (before HBV vaccines had started in South Africa). At the time of screening, the median gestational age was 22 weeks (IQR = 16–30 weeks), and 39% were < 21 weeks pregnant, a third were primigravid (33%). Almost two-thirds of women were unmarried or not cohabiting with their partner (64%). One-third of women were classified as lowest socio-economic status based on their income and assets (32%).

### Hepatitis B status

There were 8 POCT and laboratory confirmed RSAT HBV cases among 1194 women. The overall prevalence of 0.67% (95% CI = 0.34–1.32%). In women born before 1995, 8 of 622 women were diagnosed with HBsAg; the prevalence was 1.29% (95% CI = 0.65–2.52%), and in women born in 1995 or after (n = 572); the prevalence was 0% (95% CI = 0.0–0.67%).

### Confirmatory HBsAg POCT validation

We confirmed the test results in 99.8% of the rapid hepatitis B surface antigen rapid tests (Determine II). Sensitivity was 100% (8/8 results: 95% CI = 68–100%). One rapid test was invalid after 15 min (control line was absent) but was HBV positive at 30 min. All of the other rapid tests (n = 1193) were concordant at 15 and 30 min after reading the results. There were two HB surface antigen negative rapid tests that were invalid in the reference laboratory (0.2%), likely because of delays in processing the blood results or an insufficient sample. Specificity, after removing those invalid results, was 100% (1172/1172; 95% CI = 99.67–100%) (Table 1). We did not have any results that were low HBsAg positive, or discrepant that required HBV viral load analysis.

**Table 1:** Concordance between rapid hepatitis B surface antigen test (Abbott II) with gold standard laboratory testing in pregnant women, Cape Town, South Africa (N = 1192)

Abbott determine 2 HBV surface antigen Rapid test (POCT)	Gold standard hepatitis B surface antigen laboratory test (RSAT)	
	Test + ve	Test − ve
Test + ve	8	0
Test −ve	0	1184
Total	8	1184
	Sensitivity: 8/8 = 100% (95% CI = 68–100%)	Specificity: 1184/1184 = 100% (95% CI = 99.67–100%)

## Discussion

Our finding of an overall 0.67% prevalence of HBsAg in HIV negative pregnant women and 1.3% in women born before 1995 adds estimates of burden of infection in women of reproductive age in the post vaccine era in urban setting in South Africa. The high prevalence in this population could have been due to not being vaccinated against HBV or due to the fact these women were older and have had a longer time to possible exposures to HPV. These results are similar to a recent evaluation of prevalence and incidence of HBV, that demonstrated that the national prevalence of 67.76 per 100,000 population in 2019. In this national study, the HBV prevalence rate was substantially lower in individuals 15–19 years versus 20 to 24 years, with higher rates in females than males [2]. Individuals aged 25–49 years had the highest HBV prevalence rates over the 5 year period, with higher rates in males than females. HBV prevalence rates differed by Province, with the highest rate in Gauteng, followed by Eastern Cape and Kwazulu-Natal, and lowest in Northern Cape [2].

Previous studies on hepatitis B in the post-vaccination era have largely been conducted in health-care facilities or from tertiary healthcare facilities, with the majority of patients drawn from one or two provinces (Gauteng and KwaZulu Natal) or were among people living with HIV in care [16–18]. National sentinel studies reported HBsAg prevalence ranging from 2.9% in HIV-uninfected pregnant women in Western Cape in 2008 to 9.4% in HIV infected patients seeking treatment and care services in South Africa [9]. The national HBsAg test positivity rate declined annually, from 9.77% in 2015 to 8.09% in 2019, with a significant strong negative association over time [2]. The prevalence of HBV in our study is lower than other studies, even in the pre-1995 cohort, which may be due to the fact that all women were not living with HIV, and our study was in a clinic that accepted healthier, younger women (older women at risk of c-section or other outcomes were referred to the hospital for birth an antenatal care).

Critical to the elimination of hepatitis B is strengthened infant vaccination coverage and interruption of vertical transmission. The time for South Africa to join other sub-Saharan African countries in introducing the HBV birth dose vaccine, is well overdue. In our study we referred participants who tested HBsAg positive to the hospital for a birth dose vaccine as it is unavailable as standard of care in primary healthcare facilities. HBV elimination targets are set at < 0.1% HBsAg seroprevalence in children < 5 years old.

At present the South African PrEP guidelines recommend routine testing for hepatitis B prior to initiating PrEP with tenofovir [19]. The cost and barrier to testing all clients for hepatitis B prior to starting PrEP may be a barrier to starting or continuing on PrEP. In a population of young women of reproductive age, screening for HBV should not be a barrier to starting PrEP, though HBV screening should be part of the national PMTCT program. Prior studies have demonstrated that PrEP can be safely provided to individuals with HBV infection if there is no evidence of cirrhosis or substantial transaminase elevation who would require longer term treatment of HBV [20]. HBV vaccination rates at screening were low globally, despite recommendations for its use, yet uptake and efficacy were high when offered. All of the infected women were bornbefore the vaccine era, and the prevalence was 0% in those born after 1995, indicating that screening for HBsAg in vaccinated populations may not be warranted.

Limitations include that this was a selection of healthy pregnant women who were not living with HIV but interested in HIV prevention interventions including PrEP. Further, these results may not be generalizable to all Provinces in South Africa, or other regions where hepatitis B vaccine has not been implemented since birth. Unfortunately, we did not analyze the HBV viral load, IgM or HBV DNA or other serological markers to assess if infection was recent or not, and to understand vertical transmission risk of HBV in pregnant women.

## Conclusion

The prevalence of HBV was very low in pregnant women not living with HIV and was only in women born before the HBV vaccine was included in the expanded vaccine program. The Determine II POCT for HBsAg showed excellent performance against the RSAT. Critical to HBV elimination is strengthened infant vaccination coverage and interruption of vertical transmission. More needs to be done to ensure South Africa implements the HBV birth dose vaccine. HBV screening should not be a barrier to PrEP initiation in young South African women.

## Data Availability

The datasets used and/or analysed during the current study available from the corresponding author on reasonable request.
